# Scientific experiments beyond surprise and beauty

**DOI:** 10.1007/s13194-023-00536-7

**Published:** 2023-08-11

**Authors:** Anatolii Kozlov

**Affiliations:** 1 Institut Jean Nicod (CNRS-EHESS-ENS), Paris, France; 2 Department of Science and Technology Studies, University College London, London, UK; 3 Department of Philosophy, University of Geneva, Geneva, Switzerland

**Keywords:** Scientific experiments, Emotions in science, Empirical philosophy, Aesthetics of science

## Abstract

Some experimental results in science are productively surprising or beautiful. Such results are disruptive in their epistemic nature: by violating epistemic expectations they mark the phenomenon at hand as worthy of further investigation. Could it be that there are emotions beyond these two which are also useful for the epistemic evaluation of scientific experiments? Here, I conduct a structured sociological survey to explore affective experiences in scientific experimental research. I identify that learning the results of an experiment is the high emotional point in the experimenting process. Thus, experimental results can be challenging, beautiful, or boring, and they can worry, amuse, make one sad, and so on. They can also drive meta-cognitive evaluations as well as motivate specific research-related actions. From this, I advance two claims: that emotions beyond surprise and disruptive beauty are epistemically useful in empirical research, and that emotions help to overcome specific challenges of reasoning about new experimental results.

**Supplementary Information:**

The online version contains supplementary material available at 10.1007/s13194-023-00536-7.

## Experimental results, surprising and beautiful

Experiments take a special place in scientific practice. They bring about a kind of ‘domesticated’ reality, and thereby, are sources of unique data and knowledge (Morgan, 2003, 2005). Depending on the purpose, experimental results can, for example, confirm or disprove an existing theory, reveal a discovery, provide explorative hints, or serve as a calibrating reference for a new experimental technique (Currie & Levy, 2019).

To pin down their special role, Morgan proposed that experiments can be distinguished by the epistemically unique surprise they bring about. She suggested that whereas models and computer simulations, due to their deductive nature, can merely surprise, experiments can truly confound (Morgan, 2005). Currie (2018) and French and Murphy (2021) opposed this exclusion and argued that simulations and thought experiments can also be a source of epistemically ‘productive’ surprise. They proposed to capture the general essence of epistemic surprise through the discontinuity of pre-existing knowledge structures with what the experiment or theorizing reveals:

A scientific investigation is potentially productively surprising when (1) results can conflict with epistemic expectations; (2) those expectations pertain to a wide set of objects [of study]. (Currie, 2018 p. 639)

Here, surprise, in a productive sense, tracks disruptive features and aspects of experimenting and theorizing and, in being so, it violates one’s epistemic expectations. (French & Murphy, 2021; Ritson, 2020).

In a similar vein, Ivanova (2022) attributes a sense of epistemic disruption to some notable instances of aesthetic evaluation of experiments exercised by scientists. She suggests that epistemic disruption, along with experiences of wonder, awe, and mystery, might guide scientists to evaluate the limits of their knowledge regarding the experimental results and suggest some further lines for investigation (Ivanova, 2022; see also: Arcangeli & Dokic, 2020; Ivanova, 2021). However, beauty in science is not solely tied to disruptiveness. Ivanova contrasts ‘disruptive’ types of experiments with other, more aesthetically pleasing experiments. Such experiments, she suggests, may be beautiful in virtue of simplicity and clarity, with which they deliver their point; it also may be related to the efficacy, with which some crucial experiments confirm certain hypothesis (Ivanova, 2021, 2022). More generally, the importance of beauty for experiments goes in line with the role of aesthetic values in the theoretical domain of scientific research. Over the past years, several significant works have put a spotlight on scientists’ judgments of beauty about theories, models, and thought experiments (McAllister, 1999; Ivanova, 2020; Breitenbach, 2020; Currie, 2021; Brickhouse-Bryson, 2020; Murphy, 2023). The general conclusion of these studies is that aesthetic values can carry a particular epistemic weight, either, for example, in the form of useful context-specific heuristics or as contributors to attaining understanding via theories, models, and thought experiments.

If surprise and beauty are proper epistemic or cognitive categories to evaluate scientific research, what can be said about other kinds of emotions? To this moment, philosophers of science, for the most part, prudently disregarded the question of emotions, as those commonly considered problematic to the rationality and objectivity of science. Admittedly, this view is not shared by everyone. For example, virtue epistemologists provide an alternative picture of emotions. According to virtue epistemologists, knowledge is an epistemic success achieved by a skillful agent through the exercise of epistemic virtues. Epistemic virtues are either cognitive faculties (e.g., memory, perception, and introspection) or character traits (e.g., intellectual courage, open-mindedness, and intellectual autonomy) (Sosa, 1993; Greco, 2000, 2002; Zagzebski, 1996, 2013). Furthermore, emotions that are directed at the acquisition of new knowledge and understanding—so-called ‘epistemic emotions’—are also a part of this virtue ‘toolbox’ (Stocker, 2004; Morton, 2010; Candiotto, 2017). These could include, for example, curiosity, wonder, surprise, and awe (Brun & Kuenzle, 2008; Morton, 2010; Valdesolo et al., 2017; Gottlieb et al., 2018; Cuzzolino, 2021; Candiotto, 2019a, b). It is argued that epistemic emotions can constitute a motivational force, guide attention toward salient aspects of the problem, and possibly give access to otherwise unreachable facts or beliefs (Brun & Kuenzle, 2008; Candiotto, 2017, 2020; Brady, 2013; Elgin, 2007). By extension, epistemic emotions are assumed to work in science, but how exactly they do so is rather an open question. So far, virtue theory was used to explore problems of scientific underdetermination, theory choice, and questions of scientific realism (Stump, 2007; Ivanova, 2010; Baehr, 2011; Paternotte & Ivanova, 2017; Ratti, 2021; Tulodziecki, 2021). The question of emotions is still hanging in the air. To what extent emotions as virtues are indeed operative in science? And should those virtues be attributed to scientific products, to science practitioners, or to both? (Ratti, 2021).

The specific challenge of investigating emotions in science is that they rarely find their way into scientific publications and textbooks. Indeed, scientific writing is usually plain, simple, and devoid of affective expressions. Moreover, emotions are often not well regarded by scientists themselves (e.g. see the qualitative study of Stuart (2019)^1^), let alone philosophers of science (Kochan, 2013, 2015). However, there seems to be an obvious inconsistency between scientists’ declared attitudes and the actual scientific practice; indeed, many scientists suggest that their research is pervaded with various emotions and affective experiences (Wolpert & Richards, 1988, 1997; Thagard, 2002; Birney, 2013; Koppman et al., 2015). As Wolpert and Richards (1997) insist, “Scientists think and feel about their work using the same physiological apparatus as the rest of us” (p. 1). Does this mean that scientists are unable to avoid experiencing emotions in their practice? And is it indeed desirable to disregard emotional impact? Does such an impact have only a negative side? Whatever the answers to these questions are, emotions in science deserve more philosophical attention than they currently receive.

Given the little presence of emotions in scientific output, an advantageous strategy for studying emotions in science would be to directly engage with practicing researchers. Unfortunately, as far as I am aware, there are not many such empirical studies in the philosophy of science either (the few examples are: (Osbeck & Nersessian, 2011, 2012, 2013; Stuart, 2019; Thagard, 2002)).

Here, using a structured sociological survey, I explore the presence and role of emotions in the practice of experimental research. In what follows, I will outline the core content of my questionnaires (Section 2) and briefly present the main results (Section 3). In Section 4, I argue that a variety of emotions—and not just surprise or beauty—may have epistemic value for scientific experimentation. I show that the repertoire of emotion-related values can have a special role in the iterative process of empirically based reasoning, while affects, caused by acquaintance with the new experimental results, provide the salience and motivation to overcome specific challenges of reasoning regarding the results.

## Methods

### Ethics statement

This research was approved by the University Commission for Ethical Research in Geneva (CUREG-2021-08-83). Participants were provided with an ethics brief. Participation was voluntary.

### Participants

The survey was conducted in September 2021 via an online platform (LimeSurvey) and most of the participants were from the University of Geneva. It was presented to researchers who self-identify as experimentalists, that is, as researchers who ‘regularly prepare experiments, analyse experimental data, and interpret experimental results; or, as members of scientific collaboration regularly take part in the maintenance and/or preparation of experiments and have unique sets of data to analyse and interpret’.

After assessment based on the completion of all questionnaires and the carefulness of the answers, 61 responses were taken into further analysis. In total, 33 (54.1%) answers were given by males and 28 (45.9%) by female participants. Of the whole sample, 63.9% of respondents had a PhD or equivalent degree, while 34.4% had a master’s degree and 1,6% were at the bachelor’s level. Overall, the sample contained 59% of answers from biologists, 23% from physicists, 8.2% from pharmaceutical scientists, 6.6% from chemists, and 3.3% from cognitive neuroscientists.

### Design and materials

#### Questionnaire 1: Contextualisation of the overall emotional experience

**Fig. 1:**
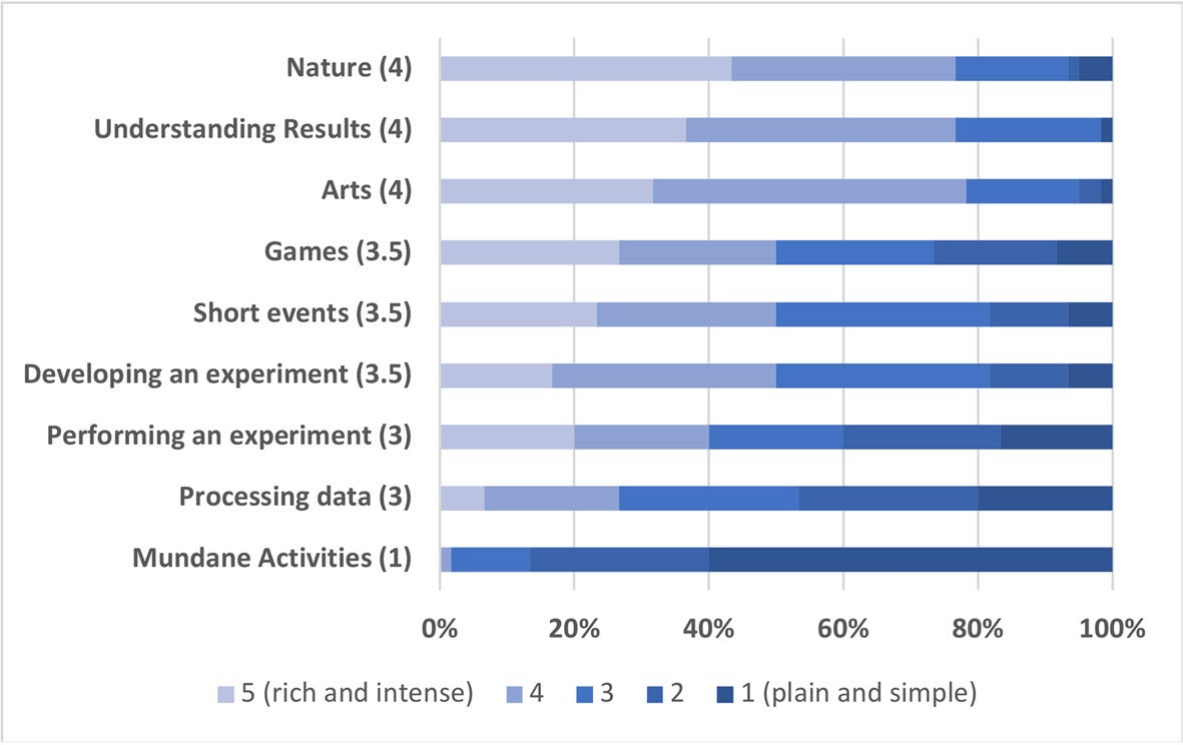
Various subjective emotional experiences on the ‘absolute’ scale. The number in parenthesis is the central tendency calculated as a median

Participants were invited to reflect upon their experience of going through different stages of individual scientific experimentation along with some other typical experiences (e.g., engagement with art, nature, mundane activities, short memorable events, and playing games (full transcript in Table S1A). Participants were asked to judge from their personal experience and estimate the average emotional experience associated with each of these activities in terms of their emotional intensity and richness (Fig. 1). The scaling was between 1 (‘plain and simple’) and 5 (‘rich and intense’). After the data was collected, the central tendency was calculated in the form of a median for each of the items.

#### Supplementary questionnaire 1: Relative ranking of emotional experiences

To supplement the outcome of questionnaire №1, the participants were subsequently invited to make a relative comparison of emotional experiences, assuming experimentation as one whole activity (from conceiving an experiment to learning and interpreting the results). The task here was to order items from the most subjectively emotionally rich and intense (top) to the least (bottom). After the data was collected, for each ranking position a percentage distribution chart was plotted (Fig. S1).

#### Questionnaire 2: Emotions in the context of confronting experimental results

Participants were invited to think about their personal emotional experience in the context of the following scenario:

(S) *Your experiment is completed, and its analysis is done. Now, for the first time, you see the full results of your experiment.*

**Fig. 2:**
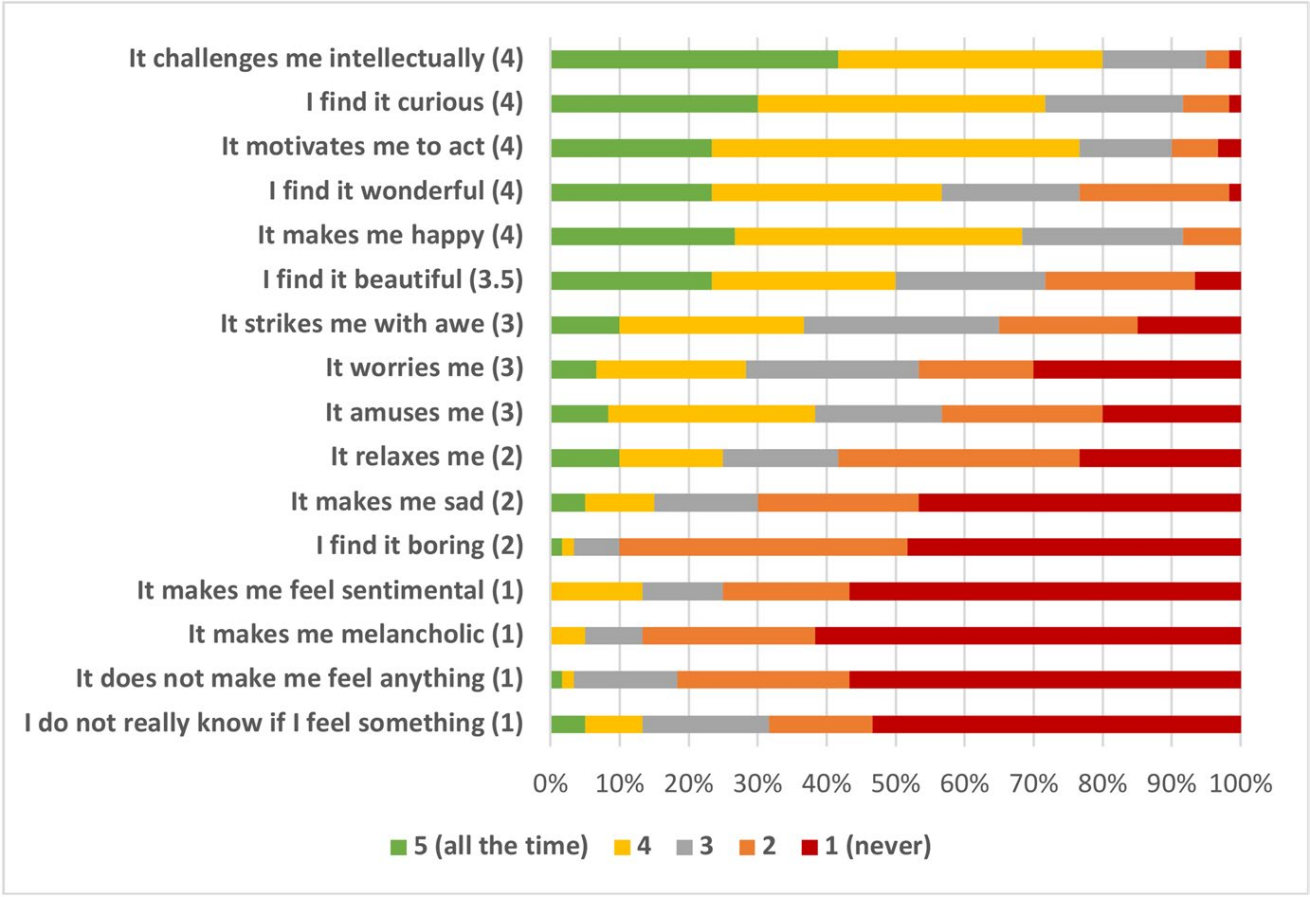
The list of emotions ordered from the most to the least frequently occurring in the context of learning experimental results. The number in parenthesis is the central tendency calculated as a median

The task was to estimate how often participants would think about their experimental result using one of the options provided in the item list (Fig. 2). The scaling was between 1 (‘never’) and 5 (‘very often’). A text box was provided at the bottom of the questionnaire in case respondents would want to share some comments in a free form. After the data was collected, the central tendency was calculated in the form of a median for each of the items.

The list of items contained a variety of emotions, supplemented by two control items that would indicate a lack of pronounced emotional experience. Thagard (2002) explored the presence of *basic* emotions within scientific research: the emotions that are apparently characterized by distinct facial expressions and are recognized universally across cultures. Building on this, the present study intended to widen the circle and test the kinds of emotions that were recently implicated in the contexts of engaging with art. Thus, the exact items for the questionnaire were selected from the AESTHEMOS survey (Schindler et al., 2017). To limit the total time for completing the survey, out of 42 items two items per type were chosen for the present study. They were ‘intellectual challenge’ and ‘curiosity’ (epistemic emotions), ‘beauty’ and ‘awe’ (prototypical aesthetic emotions), ‘amusement’ and ‘happiness’ (amusement), ‘motivation to act’ and ‘wonder’ (animation), ‘melancholy’ and ‘sadness’ (sadness), ‘relaxation’ and ‘sentimentality’ (relaxation), and ‘worry’ and ‘boredom’ (negative).

#### Questionnaire 3: Meta-cognition and action tendencies upon confronting experimental results

**Fig. 3:**
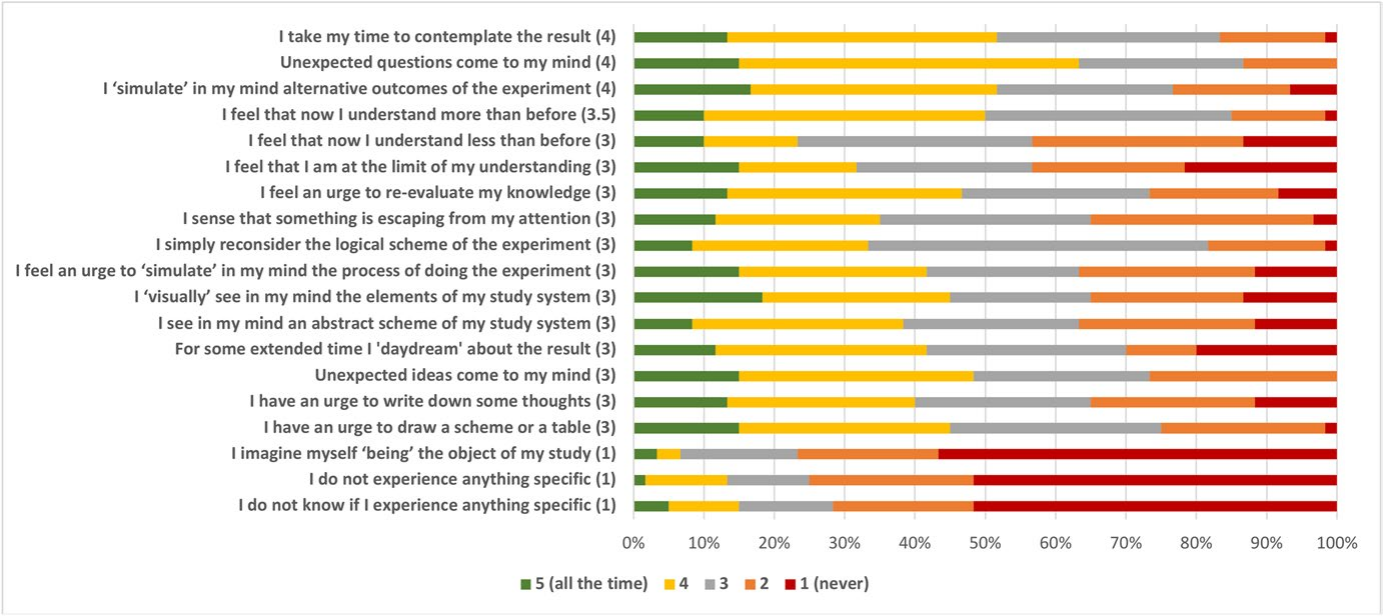
Cognitive and conative experiences. The number in parenthesis is the central tendency calculated as a median

Emotions have been implicated in supplying evaluative judgments. But in addition to that, emotions also have the capacity to motivate actions and to navigate the attention (Brun & Kuenzle, 2008; Candiotto, 2017, 2020; Brady, 2013; Elgin, 2007; Deonna et al., 2015). Whereas the previous questionnaire directly tests the presence of emotions in the context of experiments, this questionnaire explores the possible effects of emotions on researchers’ cognition and conation. Besides, just like emotions, meta-cognitive feelings, and action tendencies can have a certain phenomenological profile: they are *felt* as something. Hence, participants were invited to reflect upon some such experiences that might accompany the moment of seeing the complete result of their experiment. Participants had to choose how often, if at all, they might have one of the listed experiences. After collection, the data were processed as before (Fig. 3). At the bottom of the questionnaire, a text box was provided in case respondents wanted to share some comments in a free form.

The list of items was compiled from science-associated cognitive experiences, such as epistemic feelings. Epistemic feelings are targeted at one’s mental processes and capacities; their examples include feelings of knowing, error, familiarity, the sense of agency over thoughts, and some others (De Sousa, 2009; Arango-Muñoz & Michaelian, 2014). Deserving special attention are paradigmatic aesthetic experiences such as the experience of sublime and beautiful, which, as some suggest, contain self-monitoring components (Dokic, 2016; Arcangeli et al., 2020). In addition, items related to imaginative activities, modes of engagement with results, and thinking-assisting actions (e.g., an urge to draw a scheme or a table) were added (full transcript Table S2).

To avoid biases that may come from the item order, items in each of the questionnaires were randomly mixed for every new respondent.

## Results

### Contextualisation of emotional experience in experimental science

First, it was important to provide background for emotions in scientific experimentation. If they are indeed pronounced and salient, the participants should be able to compare their phenomenological experience in science to experiences outside of science. Thus, the comparative list contained items such as engaging with art, engaging with nature, mundane activities, short memorable events, and playing games (Fig. 1, full transcript Table S1A).

As expected, engagement with arts or nature scored the highest degree of emotional experience, while mundane activities showed the least. Games and short memorable events occupied the middle position. This was the case for both absolute evaluations of emotional experience and relative ranking (Fig. 1 and S1).

Different stages of the experimental process showed to vary emotionally from each other. While processing the data looks less engaged with emotions (mean = 3), understanding scientific results stand out as a more emotionally saturated stage in the experimental process (mean = 4). The second richest stage is the development of the experiment, which includes conceptualisation and thinking up hypotheses (mean = 3.5) while performing an experiment scored as much as the stage of data processing (mean = 3).

Interestingly, understanding scientific results turned out to be in the same group as engaging with nature and engaging with the arts, all with a mean = 4, and by a small margin, appeared to be more emotionally intense and rich than engagement with the arts (Fig. 1). This seems to be consistent with the arrangement that emerged in the relative ranking (Fig. S1), where the item of scientific experiments has the highest mode for the ranking position №3 and at the same time it shares the mode with art on the ranking position №2.

### The emotional content of confronting experimental results

Which emotions do scientists usually experience upon learning new experimental results? The goal of this questionnaire was to explore the landscape of emotions in response to reading out experimental results (see scenario (S) in Methods), and the primary focus was on their typological diversity. From the AESTHEMOS model (Schindler et al., 2017), two items per type were taken: intellectual challenge and curiosity (epistemic emotions), beauty and awe (prototypical aesthetic emotions), amusement and happiness (amusement), motivation to act and wonder (animation), melancholy and sadness (sadness), relaxation and sentimentality (relaxation), and worry and boredom (negative).

Before going further, it is worth briefly commenting on one possible limitation of this questionnaire. Not every single instance of experimentation might yield a clear-cut moment of learning some concrete result. For example, if the experiment is a small part of a large collaborative project, its interpretation would depend on the overarching result of the bigger experiment. In this case, the moment of full interpretation might be diffused in time. Indeed, a concern of this kind was expressed by one of the participants in the comment section. However, the majority of respondents belonged to research groups of 20 people or less (40%) or 10 people or less (47%), which suggests a reduced likelihood of grand collaborations in the set of answers. Besides, understanding the result of a collaborative experiment can be simply interpreted as an instance of an experiment with low emotional content. Hence, I take scenario (S) to be robust enough to dissolve this obstacle.

The summary of participants’ responses is presented in Fig. 2. It shows the distribution of items of emotion from the most (at the top) to the least (at the bottom) which frequently occur in the context of learning experimental results. This distribution clearly shows that experimental results seldom appear to be emotionally neutral or emotionally opaque. About 80% and 70% of respondents, respectively, seem to never fail or very rarely fail to have a pronounced emotional experience in response to experimental results (mean = 1). Moreover, experimental results seem unlikely to trigger melancholy or make one feel sentimental.

In contrast, epistemic emotions of curiosity and intellectual challenge scored the highest in the questionnaire, thus confirming the prominence of epistemic emotions in the scientific context. For example, 80% of respondents seem to experience intellectual challenge all the time or very often. Animation and amusement are other factors that are very common in learning about experiments: almost as often, participants seem to respond to experimental results with wonder, feeling motivated to act, and feeling happy (all mean = 4). Interestingly, aesthetic emotions such as beauty and awe are also quite common in the context of experiments; about 50% and 35% of participants, respectively, seem to experience these emotions very often or all the time. Worry, amusement, and relaxation occupied the middle position (mean = 3). While boredom and sadness were not the most frequent emotions (mean = 2), it is worth stressing that they are still a part of the landscape.

Thagard’s (2002) textual analysis explored the implication of happiness, sadness, anger, fear, disgust, surprise as well as beauty and hope in Watson’s and Crick’s famous DNA research. The present questionnaire partially overlaps with Thagard’s list and thus confirms that these emotions are present in the regular research process. It also shows that some emotions implicated in the context of art consumption are also relevant to science, the conclusion naturally complementing the results of the Q1 and SQ.

### Cognitive and conative aspects of scientific experience

The previous questionnaire showed the experimental results often motivate the respondents to act as well as to challenge them intellectually. This questionnaire explored the possible implications of such motivation and intellectual challenge. Thus, were tested the involvement of metacognitive feelings (feelings of understanding and of knowing); mental actions (various imaginative acts); and cognitive activities interpreted in terms of extended cognition. The list was also supplemented by the items that describe something like the ‘mode’ of attention to the results (contemplation, daydreaming), and by two control items associated with the lack of pronounced experiences (Fig. 3, Table S2).

Once again, the survey showed that learning scientific results tended to be associated with salient experiences rather than not (for both control items mean = 1). On the other hand, most of the items that picked up on certain experiences showed a stable frequency of occurrence. Thus, respondents seem to be quite aware of the state of their understanding or knowledge about the obtained result; they can track whether now they understand more (mean = 3.5), understand less (mean = 3), or if they reached the limit of their understanding (mean = 3). Similarly, they can spot the limit of their attention and the fact that something is escaping it (mean = 3), as well as be aware of the need to re-evaluate their knowledge (mean = 3). They also demonstrate awareness of unanticipated questions (mean = 4) or ideas (mean = 3). Contemplation of the result (mean = 4), as well as ‘daydreaming’ about it for an extended period of time (mean = 3), seem to be legitimate modes of engagement with results, which suggests the existence of mental activity targeted toward the accommodation of experimental results. What type of mental activity? One of the high-scoring items is an act of ‘simulating’ alternative outcomes of the experiment (mean = 4), a form of counterfactual reasoning. Imaginative activities, such as imagining visually the objects of study (mean = 3), as well as imagining an abstract scheme of the experiment (mean = 3), are possible, along with a simple reconsideration of the logical scheme of the experiment (mean = 3). These mental actions could be supplemented by the physical actions of writing down thoughts (mean = 3) or drawing tables or schemes (mean = 3). At the same time, imagining oneself to be the object of study is a rather unpopular strategy for thinking about experimental results (mean = 1).

## Discussion

Put together, the results of the survey suggest that the experimental process—particularly the stage of learning experimental results—commonly involves emotional experiences; they also suggest that the acquaintance with experimental results can lead to a mixed experience of an affective, cognitive, and conative nature. Such an experience to some extent is comparable to the experiences present in the context of engagement with nature and art. How robust are these results?

Most (but not all) participants of the current survey represent the same institution, while one discipline (biology) has a higher fraction of participants than the rest. Comparative analysis between the groups (biology/physics, master’s/PhD, male/female) suggests that the described results are fairly stable (Fig. S2, S3, S4). From the contextualization study (Q1, SQ), understanding experimental results remains on par with experiences of engaging with art and nature and mostly is higher than the rest stages of the process of experimentation, while sometimes (e.g., for PhDs and for males) developing the experiment reaches the same level. In terms of the content of understanding experimental results (Figs. S3 and S4), the response patterns are robust between the groups and most of the items remain in the same or neighboring relative positions as in the bulk analysis. Some slight variation in responses to control items of Q2 and Q3 suggests a possible difference in emotional sensitivity and emotional awareness between the genders, and, perhaps, between the career stages. However, the current survey is not sensitive enough to make any specific assertions about it, and more inquiry into the sociological facets of emotions in science would be interesting and useful.

The compelling aspect of the survey is that for most of the researchers, their experimental results rarely fail to trigger various affective responses. In essence, the survey suggests that experimental scientists tend to experience many different emotions in their research. I think this conclusion shouldn’t be taken as too problematic. Firstly, it balances on the verge of common sense. At the end of the day, “scientists think and feel about their work using the same physiological apparatus as the rest of us” (Wolpert and Richards (1997) p. 1). Besides, the several kinds of emotions explored here have also been observed by Thagard (2002) in his textual analysis of Watson’s and Crick’s research process. But if one would set out to cement this supposition, it can be done from a variety of perspectives; in fact, the only reason why this would not be the case is if scientists would somehow purposefully decouple themselves from their emotions, something, to my knowledge, non-existent of in the practice of science. Simply having a negative attitude towards emotions wouldn’t count as such practice as beyond the attitude there is, to my knowledge, no specific psychological or other exercises specifically tailored to prevent emotional occurrences. In all other cases, emotions as, in many ways, spontaneous reactions, would be natural to occur in the situation when one encounters the results of one’s work. The relevant questions here would rather pertain to the variability of those emotions (in terms of kinds, valences, or intensities) and to whether indeed they might have a positive contribution to the individual or communal research process and progress.

Thus, moving to more philosophical grounds, we may ask: how do emotions relate to the different epistemic outputs of scientific experiments? and can they contribute to experiment-based research?

I suggest that they do. In principle emotional values (virtues) may be attributable to both, products and practitioners (Ratti, 2021). Hence, my argument consists of two parts. First, it highlights that the repertoire of emotion-related values can have a special role in the iterative process of empirically based reasoning. Secondly, it states that affects caused by acquaintance with the newly acquired experimental results provide salience and motivation, helping to overcome specific challenges of reasoning about the results. A conclusion that follows is that a variety of emotions—and not just surprise or disruptive beauty—may have epistemic value for scientific experimentation.

### Revising productive surprise

My contention is that if surprise can be epistemically productive, it cannot be the only value in such a capacity.

Building on Morgan’s (2005) distinction between mere surprise and confoundment, Currie (2018) puts forward a case for ‘productive surprise,’ a species of surprise with a specific epistemic import. Productive surprise is bona fide epistemically good, rather than merely psychological, as it is an “occurrence which is unexpected given particular epistemic or doxastic states” (p. 645). Productively surprising experimental results can “create new phenomena, undermine old theories and hypotheses, and push investigation into unknown territory”, or, at minimum, tell us that our expectations about how the world is need re-examination (p. 656-7).

Such surprise arises from the epistemic relations between the object and the target of experimental study. The object of experimental study is a physical system in the laboratory or field: what one is experimenting on and what generates experimental data. The target of experimental study is something that the experimental system instantiates, e.g., a more general phenomenon (Parke, 2014; Radder, 2009). From this, experimental results are productively surprising when (p. 649):

1. The behaviour of the object of experimental study conflicts with doxastic or epistemic states pertaining to the object
2. Those states are externally relevant: they also pertain to the target of the experimental study and lead to changes in, or challenges to, the explanatory resources (models, theories, etc.) relevant to those states.

By narrowing our scope to externally relevant results, we must avoid the trap of being surprised by accidental results that do not have external value; for example, those that are a consequence of some contingency of the experimentation process. Indeed, the notion of productive surprise is a sub-class of the notion of surprise, which is tailored to experiments that are *successful*, where success is understood in the sense of ‘validity’ ((Currie, 2018), specifically the footnote on page 647). In cutting up the epistemic space for the surprise, Currie follows Morgan, who focuses on the fraction of experimental outcomes, that are stably reproducible yet novel:

If, after many experimental replications with many subjects and with slight variations in the experimental design, certain experimental behaviours continue to be manifest /…/, then the interpretation of the behaviour changes. It is no longer regarded as an experimental artefact and becomes a genuine behaviour pattern, and instead of trying new experimental designs to get rid of the artefact the focus becomes one of explaining the pattern. (Morgan, 2005, p. 324)

In defining what underwrites the epistemic productivity of surprising results, both Morgan and Currie rely on the view of experiments as instantiations of a ‘tamed’ reality (Morgan, 2003; Harré, 2003). Such a reality has the potential to show unexpected behaviour only if the design set-up and rules of the experiment permit a sufficient degree of freedom:

If the experimental behaviour is totally predetermined, there is no potential for unexpected patterns to emerge. Where there is no potential to exhibit alternative and unexpected behaviour, there can be no true potential to confirm theory or to refute it. (Morgan, 2005 p. 324)

It is crucial to note that Morgan talks here about experiments in economics, where the behaviour of real people is explored. Participants in these experiments, she says, if “over-tamed in a particular way”, become “agents whose behaviour is directed by models of the world, models dictated by the economist”. However, unlike experiments about economic or social human behaviour, it seems that many other types of scientific experiments are unable to attain such control over experimental objects. This might be because human participants can be directly guided by the prescriptions given in a natural language, while it is hard to see cells, particles, geological layers, or space rays being so compliant.

But there is an even stronger reason why the insufficiency of control over experimental objects is a more quotidian problem of scientific experimentation than the excess of it. Despite relying on theoretical knowledge, experiments are forms of intervention into the material world and thus are sensitive to a plethora of non-theoretical factors.

For example, the development and execution of experimental protocol (instructions on how to perform an experiment) are often shaped by pragmatic constraints (e.g., the availability of specific materials or reagents, or unanticipated or hard-to-control variations in conditions) and by factors, such as the specificity of researcher’s experimental training. Besides, research protocols often include multiple steps, while the whole procedure might span over hours, days, or weeks. Any interfering effect might not manifest itself until the final data is obtained and interpreted against the statistical noise. This means that total control over the experimental procedure is mostly unachievable: experiments often do not work out as expected due to internal factors related to the specific behaviour of the object. The following comment from the survey perfectly captures the problem:

(Quote 1) *They [results] are almost never perfect, sometimes good (and then you are very happy) and many times either bad or not so good (meaning that you still need to work on the experiment either technically or theoretically)*

How does this relate to the value of productive surprise? Recall that productively surprising experimental results must be valid (both internally and externally^2^). At the same time, the vast number of routine experiments seemingly fail to meet these standards. Yet, it would be wrong to deem these experiments epistemically useless.

What matters for learning is not only the epistemic products of a single all-encompassing experiment but the iterative progression of routine experiments accompanied by incremental doxastic changes. Researchers systematically fine-tune, recombine, and modify the existing experimental protocols, and this serves as a basis for learning.^3^ Even if experimental results fail to be internally valid (e.g., due to a flaw in experimental design), if its deviation is tractable the result can still be linked to the target phenomenon in question and therefore say something new about it. Moreover, even if the result cannot be linked to the phenomenon in question, it might point to some other phenomenon worthy of investigation. Finally, sometimes—e.g., when a new technique is being established—the experimental results are inaccessible to any extrapolation. However, they generate indispensable information about the behaviour of the experimental system itself in a particular research environment. In this case, tracking the origin of the deviation of an experimental outcome might handily disclose some previously unnoticed factors that define the behaviour of concrete experimental system. Such ‘happy chances’ are prolific sources of experimental discoveries.^4^

In sum, experimental results can be epistemically productive in more ways than productive surprise allows. Experimental results need not be perfectly valid to be epistemically useful, but their deviation from expectations, when occurs, must be tractable. Through iterative adjustments of the experimental procedure and of expectations associated with it, scientists can bring their doxastic state into close contact with the ‘domesticated’ reality of the experiment. In a more general sense, this corresponds to a simple idea that we learn from successes, but we also learn from failures, and a successful experiment is a consequence of learning from a series of failed experiments.

**Fig. 4:**
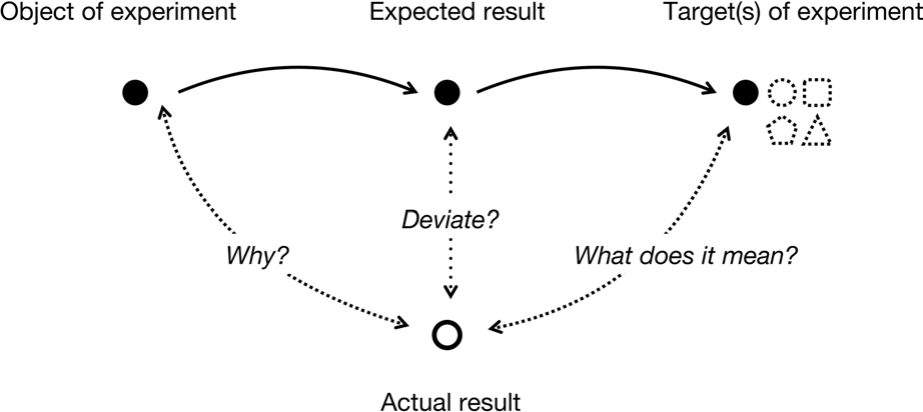
Deviation of experimental results from expectations may lead to different epistemically useful outputs

Thus, any scientifically relevant answer to why some specific results deviate from initial expectations would have some epistemic value (Fig. 4). In the next section, I will try to answer what types of values these might be.

### Emotions as values

Productively surprising results excluded experimental artefacts, which was secured by the demand that the results be valid and only concern the target of experimentation. In contrast, I argued that invalid results, e.g., artefacts of the experimentation process, can still be epistemically useful, since tracking the source of their invalidity can improve one’s doxastic state regarding the behaviour of the experimental object and/or the target. Returning to the problem of artefacts, there are two senses of understanding experimental artefacts: they can be thought of as ‘unreliable data’ (Weber, 2004); or as a ‘mistaken interpretation’ of data (Guala, 2000), and both can be summarised in the following way:

…artifacts can occur when an experimenter draws a specific inference from experimental data, not recognizing that the data do not show what the experimenter thinks they show. (Feest, 2022 p. 12)

Such inferences, in fact, are cases of misattribution. For example, if a specific theoretical hypothesis was under testing, ‘false-positive’ results corroborate it by misattributing the contingent behaviour of the experimental object to the anticipated behaviour of the target. Similarly, the ‘false-negative’ results misattribute the supposedly unanticipated behaviour of the target to the contingent behaviour of the object. False-negative results might appear surprising, while false-positive might not, however, both have the potential to challenge one’s doxastic state about the experimental system, and scientists are aware of the deep ambiguity of experimental outcomes:

(Quote 2) *There is a part of me always wondering whether the results were not biased by some unknown factor, for example, error in doing the experiment or flaw in experimental design, so urge to double-check and question the process. Another part of me is trying to find what are the implications at a larger scale of the result, so that’s where I question my own understanding and knowledge.*

Ever since Kuhn (1977) and McMullin (1982), evaluations are considered to be an inescapable part of science, specifically in the contexts of theory choice and hypothesis-making. What the present analysis suggests is that inferential reasoning is not the only thing that is going on in the experimenting process and that the experimental results prior to or in addition to inferential reasoning can or even must also be evaluated. For if we are to make inferences from experimental results, we also must assess the reliability of these results. Moreover, the evaluation of the results itself can determine the types of inferences we are making. For example, if the results support some plausible explanation but do not look trustworthy, we need to assess why exactly they don’t look trustworthy.

Multiple values are typically attributed to scientific theories, such as novelty, consistency, simplicity, accuracy, fertility, and so on (e.g., (Longino, 1996; Kuhn, 1977; Schindler, 2022)). Keas (2018) classifies theoretical values into four classes: evidential, coherential, aesthetic, and diachronic. What values can be attributed to experimental results? Some must be resembling the theoretical ones. Thus, for example, are valuable the mentioned features of intrinsic and extrinsic validity of the experimental output. Some form of novelty is tracked by epistemic surprise. In fact, the form of epistemic surprise defended by Morgan and Currie clumps together values of novelty and validity. However, there must be many more than that. There can be properties such as trustworthiness, clarity, coherence, scope, fertility, complexity, aesthetic appeal, and, possibly, others. The value space of experimental results is underpinned by the relations between three domains of reference: knowledge and beliefs about the *object*, about the experimental *procedure*, and about the target of the *experiment*. The specificity of concrete experimental results can then be picked up by different values with different epistemic repercussions and would say something particular about the object, the process, the target, or the combination of all three.

McMullin (1982) reckoned that values in science are not emotive:

It seems plausible to hold that emotive values are alien to the work of natural science. There is no reason to think that human emotionality is a trustworthy guide to the structures of the natural world. Indeed, there is every reason, historically speaking, to view emotive values, as Bacon did, as potentially distortive “Idols”, projecting in anthropomorphic fashion the pattern of human wants, desires and emotions on a world where they have no place. (p.5)

However, I suggest the contrary: bound by the context and attuned to the epistemic aims of science,^5^ emotive evaluations about experimental results *can* be a form of epistemic assessment relayed via relational knowledge. It is in this sense that disruptively beautiful or surprising results indicate their potential future fertility (Ritson, 2020; Ivanova, 2021). In addition, beauty and partucularly awe both indicate the potentially large scope and epistemic power of the results (Arcangeli & Dokic, 2020; Ivanova, 2021); curiosity might track novelty plus complexity (Schindler et al., 2017). Unexpectedly boring results may indicate a need for a more creative approach to experimenting and theorizing. On the other hand, *expectedly* boring results, e.g., those derived from multiple repeated control experiments, might cement one’s confidence in the actual frame of reasoning. Sadness may apply to the results that didn’t work in some particularly counter-productive or hopeless fashion. Intellectual challenge possibly represents a core value, admixed to most of the other kinds of epistemic evaluations of experiments.

The present survey shows that most scientists apply affective evaluations towards experimental results (Fig. 2), which, conversely, means that experimental results embody a variety of values with potential epistemic import. Additionally, the list of emotions tested here might not even be sufficiently representative, as many other emotional species might occur in this context. For example, the fact that experiments often do not work means that negatively valenced evaluations must also occur often. Indeed, some respondents’ comments indicate the presence of such entities as frustration and anxiety.

If the values are roughly coherent and shared by the community, then the supposedly subjective emotive evaluations may be inter-subjective, in the similar way how finding something beautiful, frightening or funny, on the one hand, can be shared between people and, on the other, sometimes is attributable to the stable and tractable features of the world. Perhaps, an illustrative example of such an inter-subjective evaluative term can be the so-called ‘Nightmare scenario’ in particle physics, which denotes the possibility that Large Hadron Collider won’t disclose any new physics beyond the Standard Model; whereas it refers to strictly epistemic collision, for the many involved researchers it can also impose a distinctively emotive sense (Cho, 2007; Bertone et al., 2012; White, 2014; Hossenfelder, 2018).

### Emotions and cognitive actions

Having discussed the epistemic role of emotions as values embodied by experimental results, now, briefly, I will consider emotions from a functional cognitive perspective, using the results of Questionnaire 3 (Fig. 3) as a cue. I will appeal to emotions as patterns of salience and motivation. It is these properties that prompted some epistemologists to consider emotions as epistemic values (Sousa, 1987; Brun & Doguoglu, 2012; Brady, 2013; Candiotto, 2017).

To remind, Questionnaire 2 indicate that experimental results often prompt one to experience intellectual challenge and motivation to act. Questionnaire 3 explores the dimensions of metacognition that are (plausibly) incited by intellectually challenging results as well as various cognitive acts, such as acts of counterfactual reasoning, visual imagination, acts of drawing schemes or writing something down; the latter two can be considered as cognitive in an extended sense: writing things down helps to free up working memory, reconfiguring attention, and relaying reasoning through external aiders.

Now, let us briefly return to the case of surprising results: results that contradict certain epistemic expectations. To become a case of learning, such a state needs to be resolved; we need to track the source of the surprise and revise our epistemic state concerning the source. The point is that the outcome of a particular experiment, namely, the behaviour of the object of experimentation, can be explained by appeal to three different explanatory sources: properties of the target of the experiment, properties of the object of experimentation (that do not pertain to the target), and properties of the experimental performance. The three sources are mutually informative; for example, learning that the result is a product of a performative accident, in principle, refutes an explanation from the target. At the same time, all three sources are epistemically heterogeneous, requiring one to have not only theoretical knowledge (‘know-why’) but also descriptive and case-based knowledge about behaviours of the object in various conditions (‘know-that’), as well as a thorough theoretical and practical understanding of the processes and instruments involved in the performance of experiments (‘know-how’). This puts two specific challenges to any attempt to explain experimental results: the challenge of the abundance, and the challenge of the heterogeneity of the explanatory resources.

The first challenge is a particular case of the framing problem. Looking at fresh results, one can be puzzled about which interpretative framework to adopt; something even more important given that different interpretations invite different inferential strategies. As Sousa (1987) pointed out, “important areas of indeterminacy have to do with what subjects to investigate, and what inductive rules to adopt” (p. 191). The problem is that “no logic determines salience: what to attend to, what to inquire about”, so emotions can be useful here. Taken as values, emotions serve as heuristics about what epistemic import the given results may bring. However, considered functionally, they can do this by providing patterns of salience concerning the specificity of given results in relation to the available explanatory sources. Thus, patterns of attention that come along with phenomenal experience about certain experimental results identify in them something that does (or does not) look ‘right’, does (or does not) seem to ‘fit’, and by these means spotlight the areas in one’s doxastic state that are of potential relevance for solving the problem (Brun & Kuenzle, 2008).

This also links to the second problem: the heterogeneity of the relevant explanatory resources, and by extension, the heterogeneity of the inference strategies one has at hand. What looms here is that there is no single algorithm or strategy for how one should evaluate experimental results. Instead, one can expect a plurality of methods and devices that help bring different explanations under some common denominator. Indeed, the survey (Fig. 3) shows exactly this: a plurality of mental and physical actions can be summoned by the acquaintance with the result. Some examples are imagining visually, simulating mentally, imagining counterfactually, reasoning logically or diagrammatically, recalling semantically or episodically, engaging in extended episodes of perception or daydreaming, as well as evaluating meta-cognitively one’s efforts to accommodate results. Something should incite and perpetuate this activity, and here the motivating aspect of emotions cannot be downplayed. Some philosophers specifically take emotions to be states of evaluative action-readiness (Scarantino, 2014; Deonna & Teroni, 2015). Such interpretation may not be consensual, yet it is generally uncontested that emotions have a close connection to actions. Such a connection is significant science. In the famous chapter on epistemic emotions, Adam Morton (2010) paints an image of “an extraordinarily well-trained and malleable young scientist”, who “does not care about the subject”:

She does not feel wonder at the connections between facts that she can glimpse through the data. She does not feel curiosity about what scientists two hundred years later will have arrived at. Nor does she feel momentary skepticism — in everyday language a loaded attitude rather than a philosophical position — about whether current techniques can unlock the further secrets of the topic. (p. 389)

For such a researcher, he says, “the absence of epistemic emotions seems to make things harder”. Emotions are a key to persistent motivation “to follow up lines of inquiry to their ends and develop new lines when the ones we have followed have got to unsatisfying ends”. From the results obtained here, we can also add that the motivating force of emotions might be regulating science in the day-to-day micro-level of experimental research. Through patterns of salience, they motivate specific research strategies in response to specific empirical challenges, acting like ‘railroad switches’ between them. Thus, in worrying that the results might have been obstructed by an accident in the performance, we scan our episodic memory associated with the execution of the protocol, counterfactually simulate what deviation in the process may have caused the specific outcome or check the physical intactness of the instruments. Alternatively, confident in the results, we may wonder about their effect on another domain of knowledge; we may spontaneously devise a thought experiment that puts the two domains into contact, and be further productively surprised by the output of such an exercise (French & Murphy, 2021). The very plasticity of affective response might be what links the variability of experimental results with the plurality of cognitive and epistemic tools used to evaluate and cognitively accommodate them.

Saying all of this, the potential negative impact of emotions on experiment-based reasoning cannot be discounted. Sometimes, emotions can indeed allow the desire or will override the reason or perception – or ‘skew the epistemic landscape’ in some other sense (Goldie, 2008; Wild, 2008). More than that, as some survey respondents’ comments suggest, doing experimental research can be a ‘rollercoaster of emotions’, while when an experiment fails ‘it can be very frustrating and affect our well-being negatively’. However, these reasons, I believe, must convince us that emotions should be considered with serious attention rather than dismissed as simply irrelevant to science, scientific process, and scientific progress.

## Conclusions

In the present work, I sought to investigate emotions in the context of experimental research. By conducting a structured sociological survey, I showed that experimental research, particularly the stage of acquaintance with new experimental results, is enriched with various emotional experiences. Further, I defended the role of emotions as values in relation to experimental results, suggesting that they can represent genuine epistemic values of experimental results accessed partially via relational knowledge. In addition, I showed the importance of emotions from the functional perspective, suggesting that their capacity to navigate attention and to motivate action drives the execution of specific epistemic strategies that help to evaluate and to accommodate newly obtained experimental results.

### Supplementary Information

ESM 1 (DOCX 9.68 KB)

Fig. S1 (PNG 1624 kb)

High resolution image (TIF 3556 kb)

Fig. S2 (PNG 228 kb)

High resolution image (TIF 1328 kb)

Fig. S3 (PNG 362 kb)

High resolution image (TIF 2387 kb)

Fig. S4 (PNG 1344 kb)

High resolution image (TIF 2482 kb)

## Footnotes

1 A quote: “Finally, I asked participants the same question concerning the role of emotion in their work. Here there was slight variability: The more senior a participant was, the more roles they could identify for emotion in their work. Nevertheless, even comparing the most to least senior lab members, none of them attributed any positive epistemic role to emotion. Rather, they treated it as a danger to be avoided”. (p. 720)

2 An experiment is internally valid when instruments work properly and experimental design works as intended; an experiment is externally valid when we expect that experimental results are projectable onto the target. (Currie, 2018, p. 652)

3 In fact, iterative epistemic bootstrapping seem to be a feature of both experimental *and* model-based research (Chang, 2007; Nersessian, 2010; Currie, 2017)

4 As a story circulating among biologists goes, there were two postdocs in the lab. The diligent ‘early bird’ would extract RNA of a gene early in the morning; the more relaxed one would come to the lab much later and start RNA extractions of the same gene sometime in the afternoon. As the second postdoc would always fail to get the RNA, the suspicion was that this postdoc needed to buckle down and get more serious about the work. But as it turned out later, the gene in question simply followed a circadian pattern of transcription and was barely transcribed in the afternoon.

5 See (Currie, 2021) on the attunement of aesthetic values in science.
