# Supplementary material for: Scientific experiments beyond surprise and beauty
Source: Eur J Philos Sci. 2023 Aug 11;13(3):38. doi: 10.1007/s13194-023-00536-7 (PMC10415511; doi:10.1007/s13194-023-00536-7)
Supplement: Supplementary file 1 — (DOCX 9.68 KB) [file 13194_2023_536_MOESM1_ESM.docx]

**Supplementary Materials**

**Table S1A - Subjective evaluation of emotional experience, item list**

| **Activity** |
| --- |
| Engaging with arts (e.g., watching a movie, listening to music, visiting a gallery, watching a theatre play) |
| Engaging with nature (e.g., visiting parks, rivers, mountains, seaside, etc.) |
| Playing games (e.g., board games, sports games, computer games, etc.) |
| Mundane activities (e.g., household chores, commuting to the job, filling bureaucratic forms, etc) |
| Short memorable events (e.g., hearing an anecdote, receiving a call from a friend, writing a complaint, noticing a scary insect, forgetting your phone at home) |
| Developing a scientific experiment (e.g., conceptualising the framework, establishing the experimental protocol) |
| Performing a scientific experiment (e.g., preparing the necessary tools, executing the experimental protocol) |
| Processing experimental data (e.g., doing technical analysis of images, quantifying the statistics) |
| Understanding experimental results (e.g., seeing the final data for the first time, interpreting the outcome, making connections) |

**Table S2 Feelings and actions**

| **Experience** |
| --- |
| For some extended time, I ‘daydream’ about the result |
| I feel an urge to ‘simulate’ in my mind the process of doing the experiment |
| I feel an urge to re-evaluate my knowledge |
| I feel that now I understand more than before |
| I feel that now I understand less than before |
| I feel that I am at the limit of my understanding |
| I sense that something is escaping from my attention |
| I ‘visually’ see in my mind the elements of my study system (e.g., particles, cells, waves) |
| I see in my mind an abstract scheme of my study system |
| I ‘simulate’ in my mind alternative outcomes of the experiment |
| I imagine myself ‘being’ the object of my study (I imagine the world from the point of view of this object) |
| Unexpected ideas come to my mind |
| I take my time to contemplate the result |
| Unexpected questions come to my mind |
| I have an urge to write down some thoughts |
| I have an urge to draw a scheme or a table |
| I simply reconsider the logical scheme of the experiment |
| I do not experience anything specific |
| I do not know if I experience anything specific |

**Anonymized data of responses to the survey can be found here:**

Kozlov, Anatolii, 2023, "Scientific experiments: a survey", <https://doi.org/10.7910/DVN/KTWY82>, Harvard Dataverse, V1
