# Supplementary figures and images for: Scientific experiments beyond surprise and beauty
Source: Eur J Philos Sci. 2023 Aug 11;13(3):38. doi: 10.1007/s13194-023-00536-7 (PMC10415511; doi:10.1007/s13194-023-00536-7)

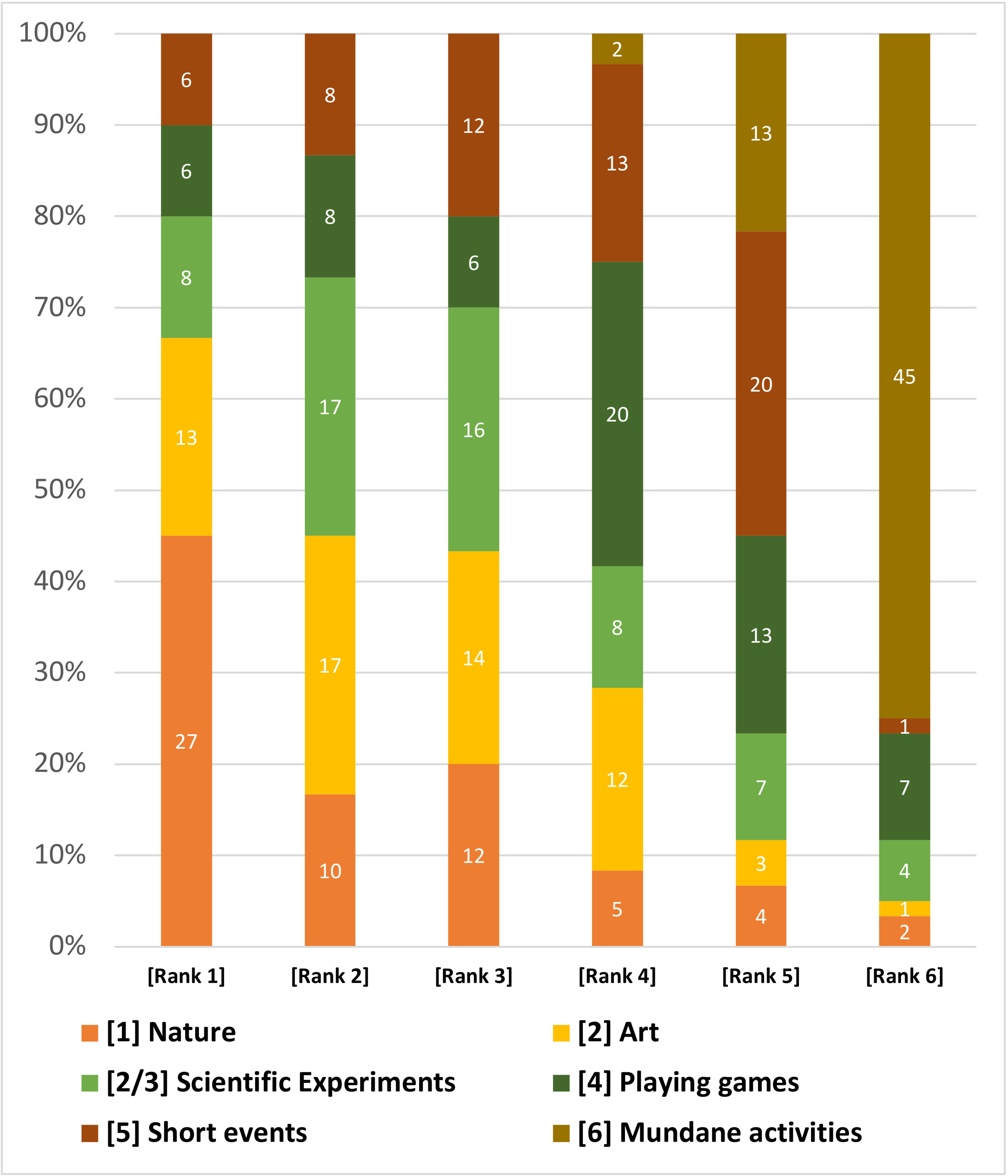

Supplement: Supplementary file 2 — (PNG 1624 kb) [file 13194_2023_536_Fig5_ESM.png]

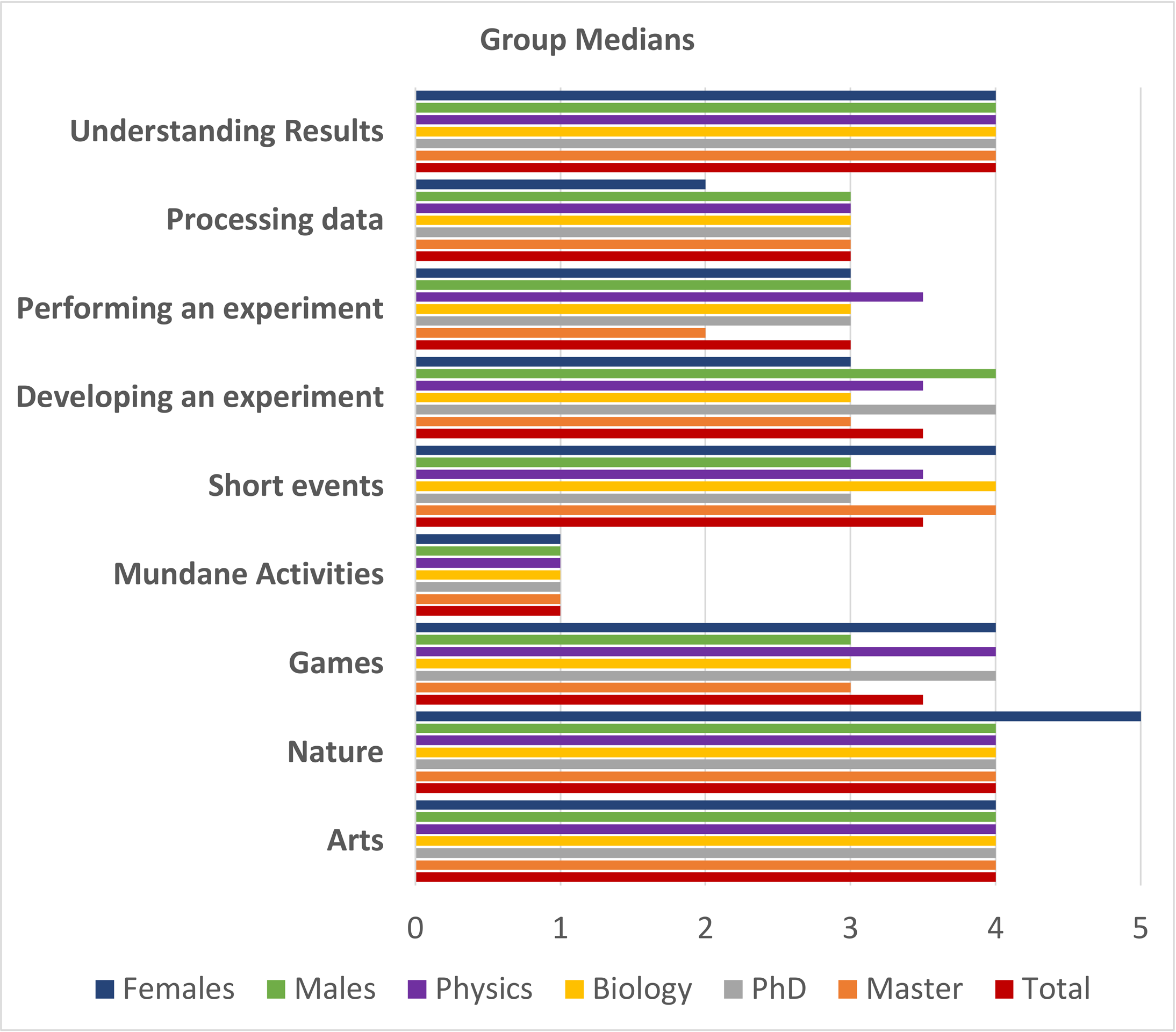

Supplement: Supplementary file 4 — (PNG 228 kb) [file 13194_2023_536_Fig6_ESM.png]

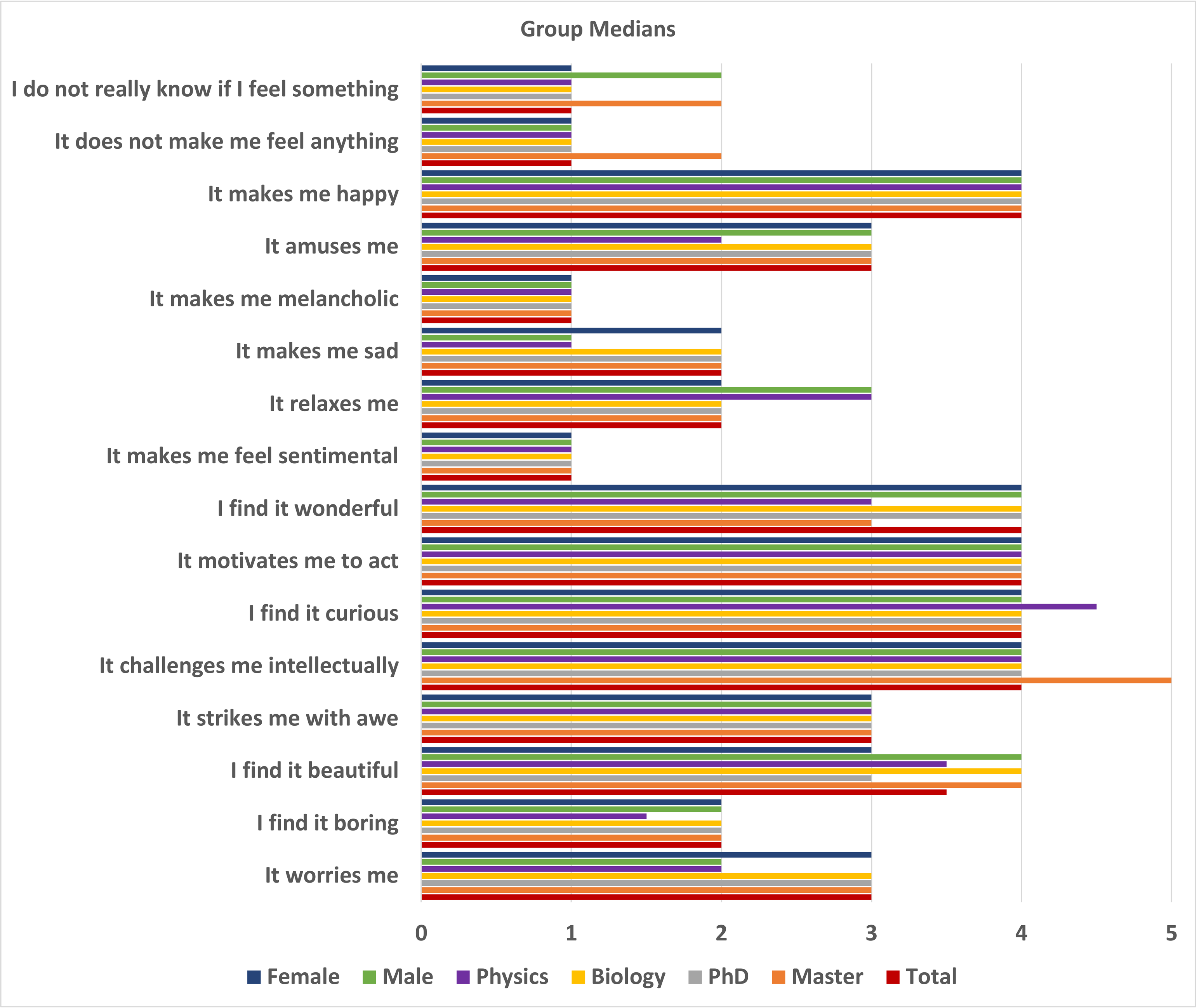

Supplement: Supplementary file 6 — (PNG 362 kb) [file 13194_2023_536_Fig7_ESM.png]

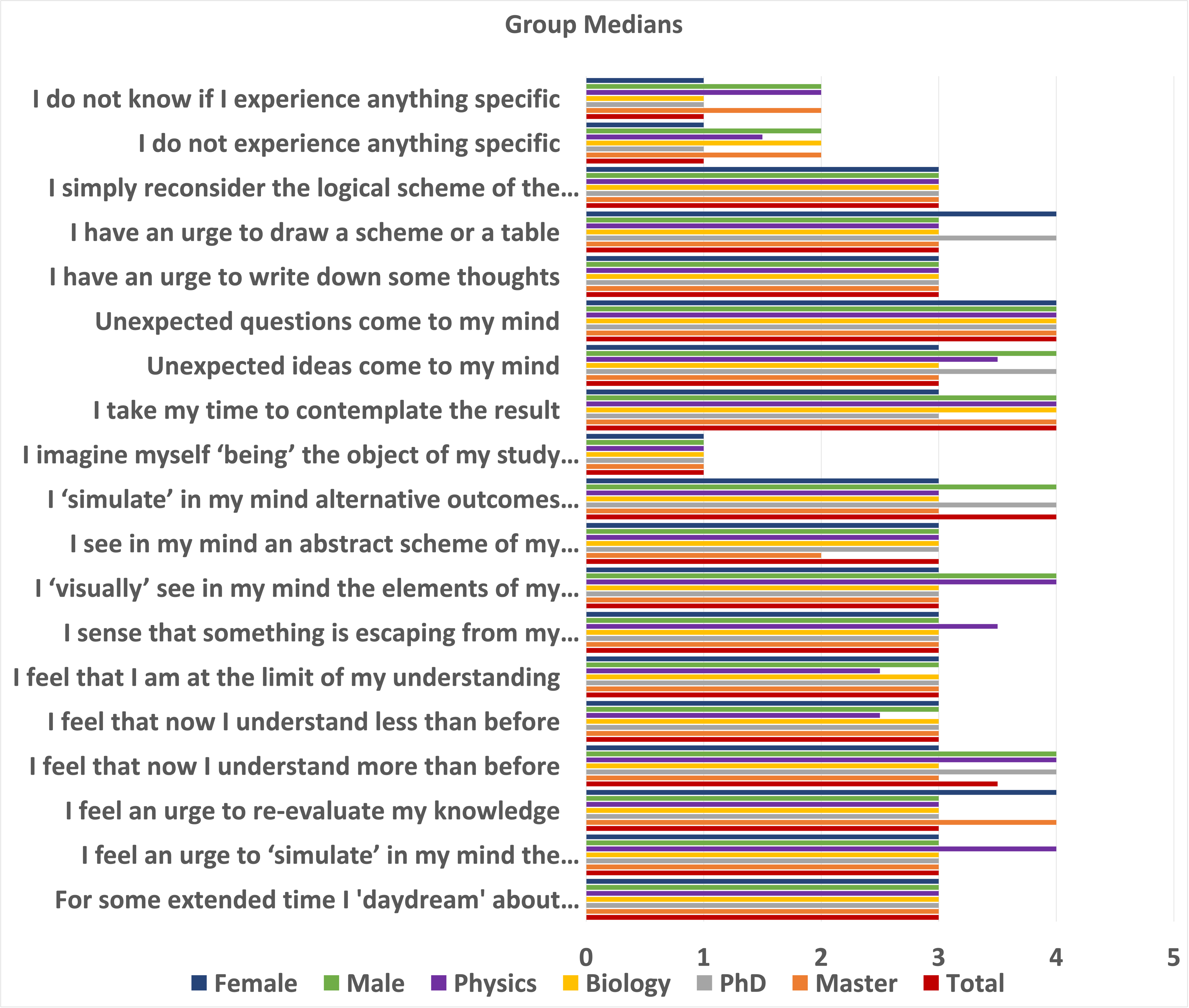

Supplement: Supplementary file 8 — (PNG 1344 kb) [file 13194_2023_536_Fig8_ESM.png]

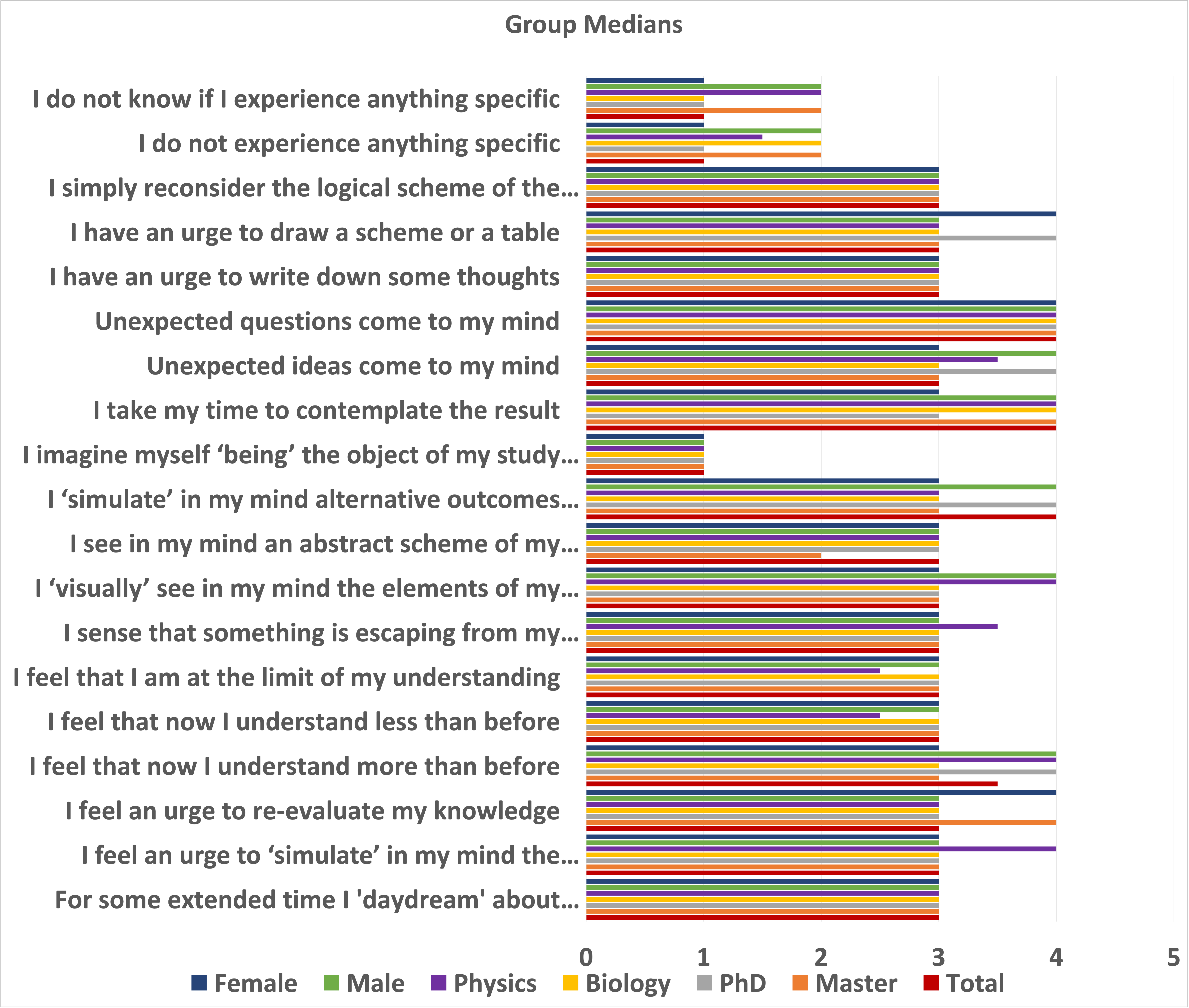

Supplement: Supplementary file 9 — High resolution image (TIF 2482 kb) [file 13194_2023_536_MOESM5_ESM.tif]
